# Supplementary material for: Structural dynamics of the active HER4 and HER2/HER4 complexes is finely tuned by different growth factors and glycosylation
Source: bioRxiv. 2024 Jan 4:2023.10.06.561161. Preprint. [Version 2] doi: 10.1101/2023.10.06.561161 (PMC10802258; doi:10.1101/2023.10.06.561161)
Supplement: 1 [file NIHPP2023.10.06.561161V2-supplement-1.pdf]

bioRxiv preprint doi: https://doi.org/10.1101/2023.10.06.561161; this version posted January 4, 2024. The copyright holder for this preprint (which was not certified by peer review) is the author/funder, who has granted bioRxiv a license to display the preprint in perpetuity. It is made available under aCC-BY 4.0 International license.

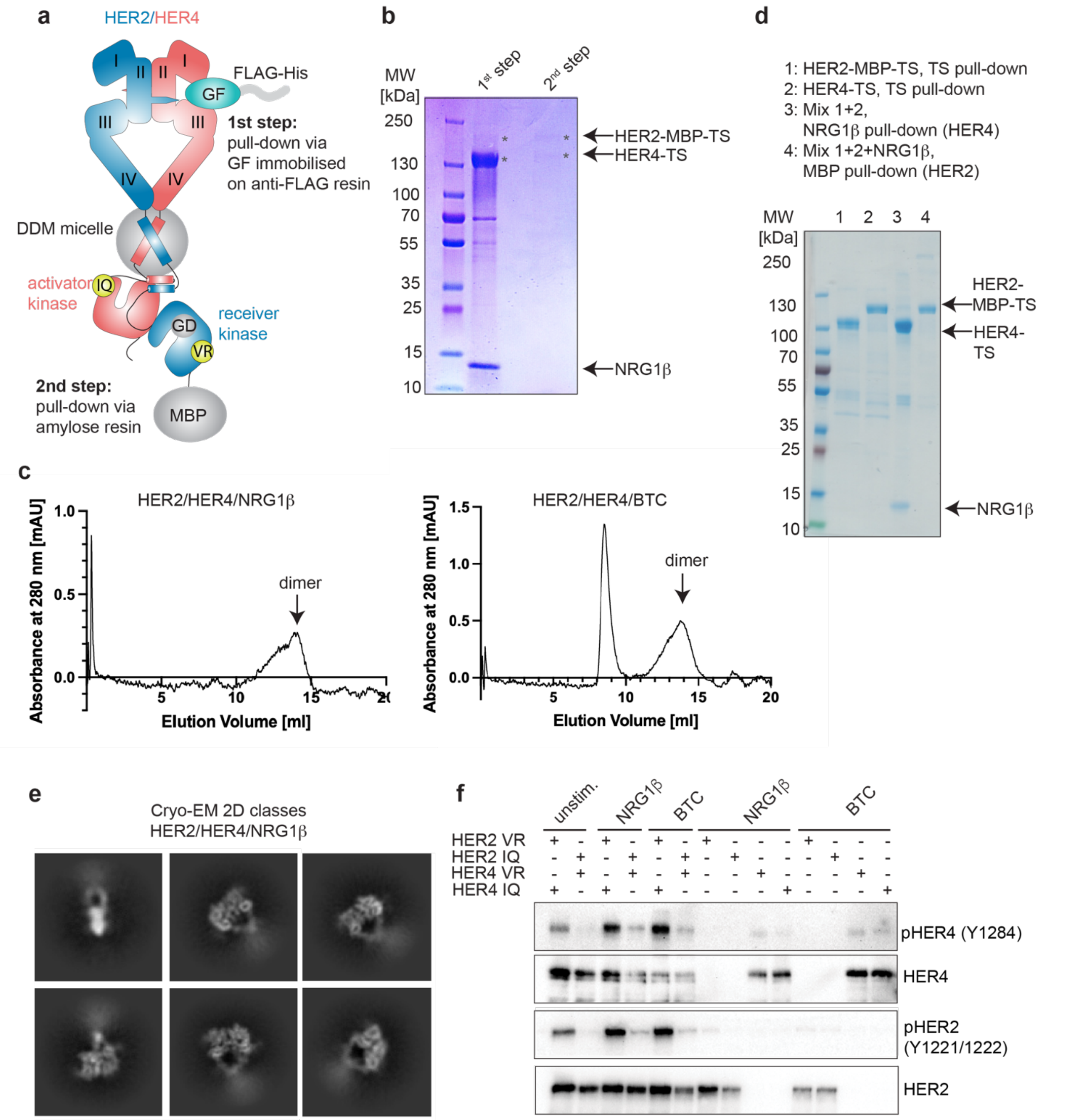

Figure S1: Purification and the functional analysis of the HER2/HER4 heterodimers. **a**, Overview of the HER2/HER4 purification strategy. HER2 features a G778D (GD) mutation to mediate Hsp90-independence. VR corresponds to HER2-V956R (receiver) and IQ to HER4-I712Q (activator). The mutant complex was used for all purification and structure determination steps shown in this figure (panels b-e) and is referred to as HER2/HER4. **b**, Coomassie-stained SDS-PAGE gel analysis of the samples from the HER2/HER4 purification after ligand-mediated

bioRxiv preprint doi: https://doi.org/10.1101/2023.10.06.561161; this version posted January 4, 2024. The copyright holder for this preprint (which was not certified by peer review) is the author/funder, who has granted bioRxiv a license to display the preprint in perpetuity. It is made available under aCC-BY 4.0 International license.

pulldown (1st step) and MBP pulldown (2nd step). **c,** Representative Size Exclusion Chromatography (SEC) profiles for liganded HER2/HER4 heterocomplexes. **d**, Coomassie-stained SDS-PAGE gel analysis of indicated HER2 and HER4 pulldown experiments. Lanes 1 and 2 show HER2-MBP-TS and HER4-TS TS (Twin-Strep) pulldown eluates. Eluates from lane 1 and 2 were mixed and NRG1β-mediated (lane 3) or MBP pulldowns (amylose resin, lane 4) were performed **e**, Representative 2D cryo-EM class averages of liganded HER2/HER4/NRG1β heterocomplexes. Box size is 321 Å. **f**, Western Blot showing that activation of HER2/HER4 heterodimers requires HER2 to adopt the kinase receiver function (HER2-VR) and HER4 to adopt the kinase activator (HER4-IQ) function in the heterodimer. Full-length constructs were co-transfected into COS7 cells, starved overnight and stimulated with 10 nM ligand for 10 min at 37 °C. The HER2 constructs used in this experiment do not feature the G778D mutation. The blot is representative of three independent experiments.

bioRxiv preprint doi: https://doi.org/10.1101/2023.10.06.561161; this version posted January 4, 2024. The copyright holder for this preprint (which was not certified by peer review) is the author/funder, who has granted bioRxiv a license to display the preprint in perpetuity. It is made available under aCC-BY 4.0 International license.

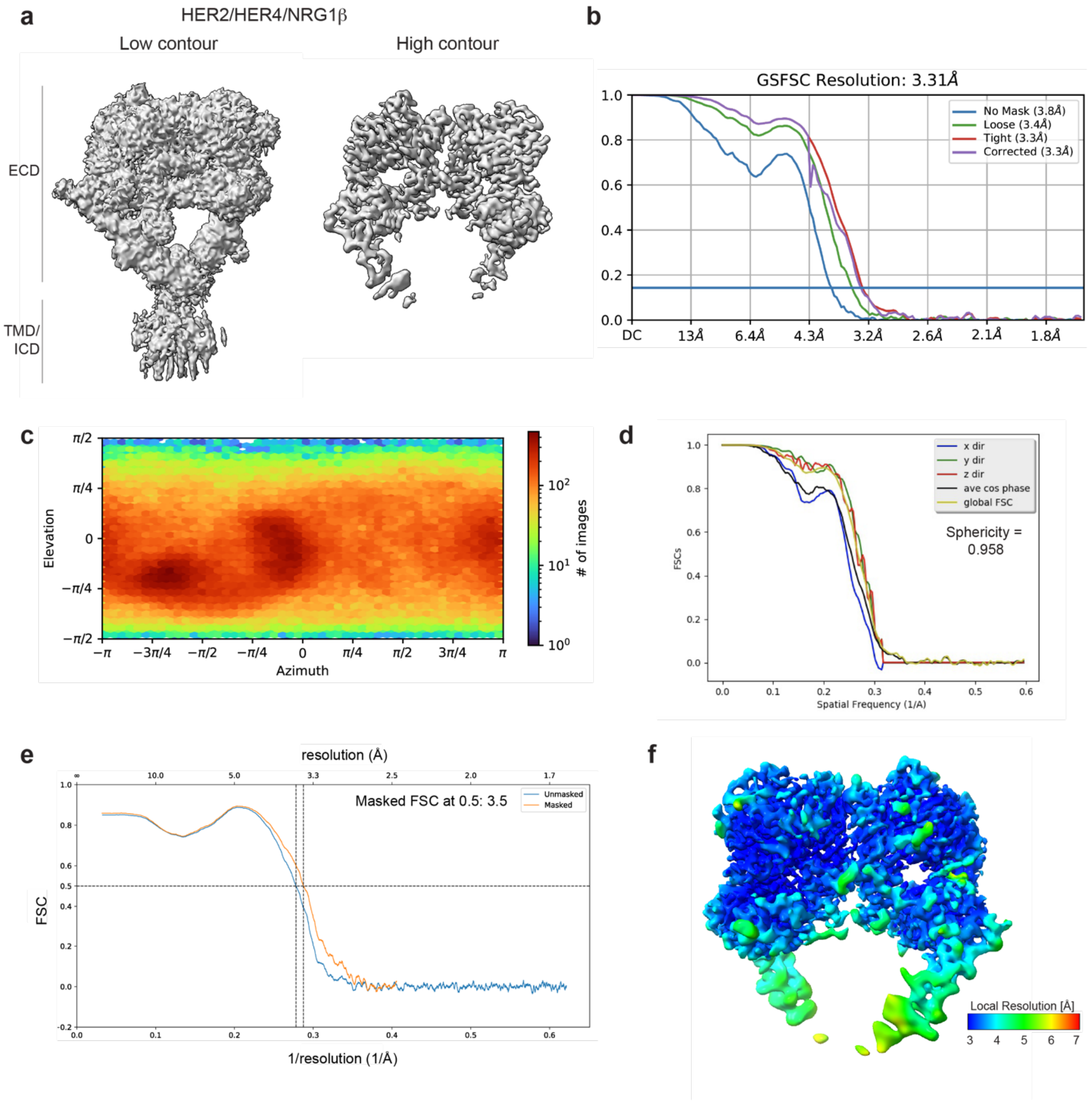

Figure S2. Cryo-EM density maps of HER2/HER4 bound to NRG1β. a, Cryo-EM map at different contour levels. **b,** CryoSPARC GSFSC plots. **c**, CryoSPARC Euler angle plots. **d**, 3DFSC plots. **e,** Model-Map-FSC curves from Phenix Validation **f,** Local resolution map of HER2/HER4/NRG1β created using cryoSPARC v4.

bioRxiv preprint doi: https://doi.org/10.1101/2023.10.06.561161; this version posted January 4, 2024. The copyright holder for this preprint (which was not certified by peer review) is the author/funder, who has granted bioRxiv a license to display the preprint in perpetuity. It is made available under aCC-BY 4.0 International license.

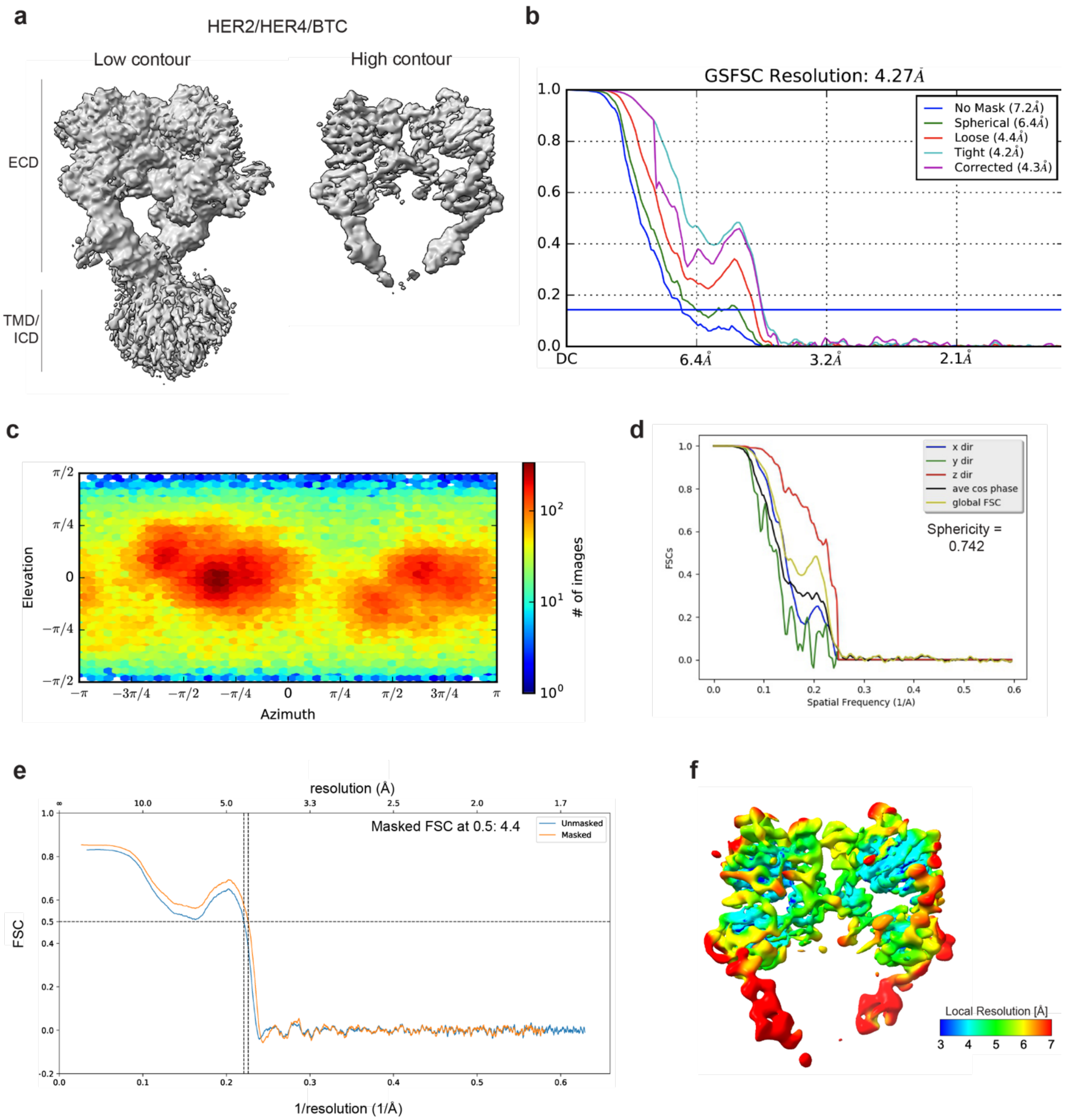

Figure S3. Cryo-EM density maps of HER2/HER4 bound to BTC. a, Cryo-EM map at different contour levels. **b,** CryoSPARC GSFSC plots. **c**, CryoSPARC Euler angle plots. **d**, 3DFSC plots. **e,** Model-Map-FSC curves from Phenix Validation **f,** Local resolution map of HER2/HER4/BTC created using cryoSPARC v4.

bioRxiv preprint doi: https://doi.org/10.1101/2023.10.06.561161; this version posted January 4, 2024. The copyright holder for this preprint (which was not certified by peer review) is the author/funder, who has granted bioRxiv a license to display the preprint in perpetuity. It is made available under aCC-BY 4.0 International license.

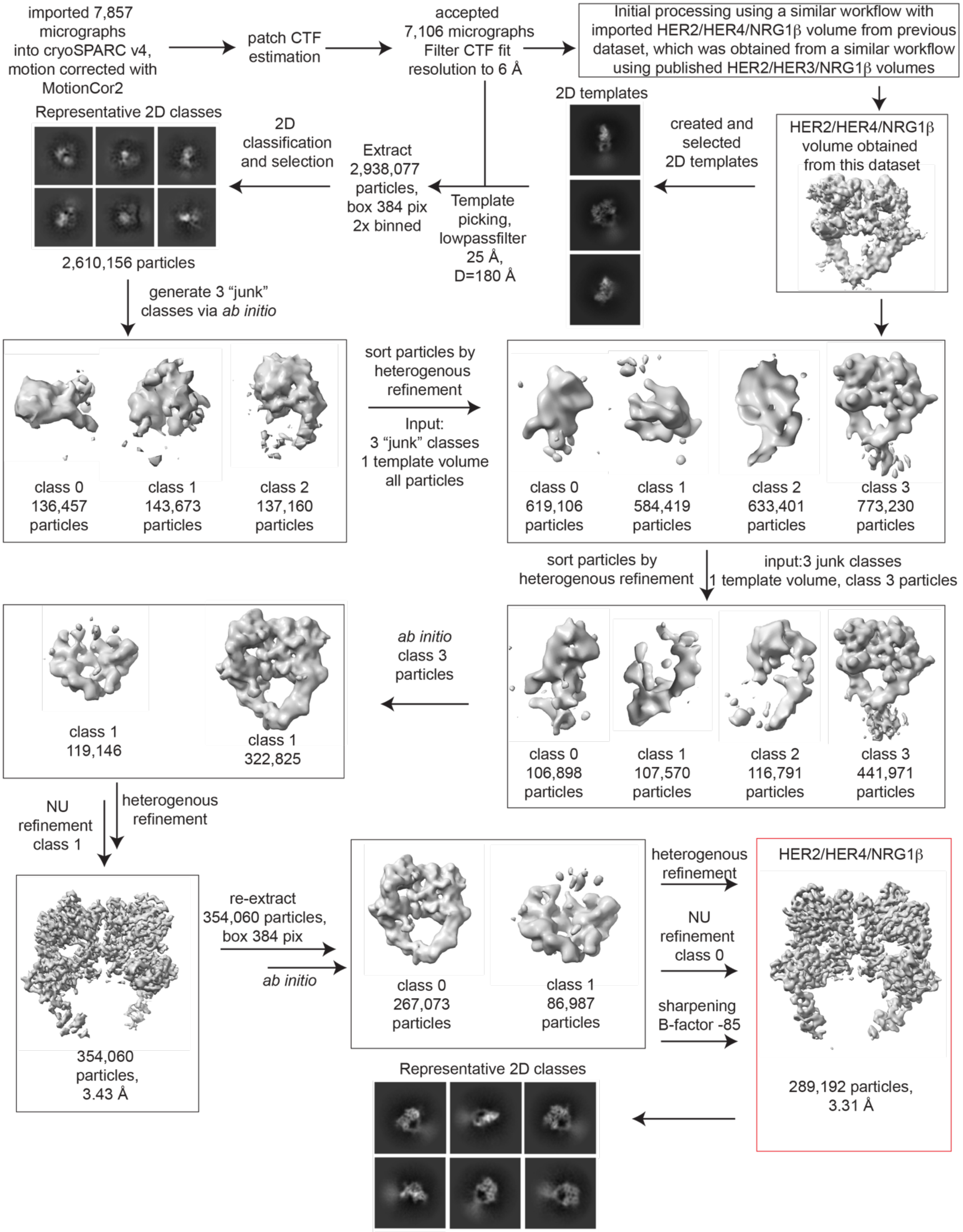

Figure S4. **Processing workflow for the HER2/HER4/NRG1β structure**. Data were processed in cryoSPARC v4 using a strategy in which particles are picked generously using template picker,

bioRxiv preprint doi: https://doi.org/10.1101/2023.10.06.561161; this version posted January 4, 2024. The copyright holder for this preprint (which was not certified by peer review) is the author/funder, who has granted bioRxiv a license to display the preprint in perpetuity. It is made available under aCC-BY 4.0 International license.

selected by 2D classification to remove bad picks (<10% of particles) and then sorted via 2 rounds of heterogenous refinement into a HER receptor dimer template volume and 3 “junk” classes created from the impure particle stack. Picked particles were subjected to *ab initio* reconstruction to eliminate bias and further processed as shown.

bioRxiv preprint doi: https://doi.org/10.1101/2023.10.06.561161; this version posted January 4, 2024. The copyright holder for this preprint (which was not certified by peer review) is the author/funder, who has granted bioRxiv a license to display the preprint in perpetuity. It is made available under aCC-BY 4.0 International license.

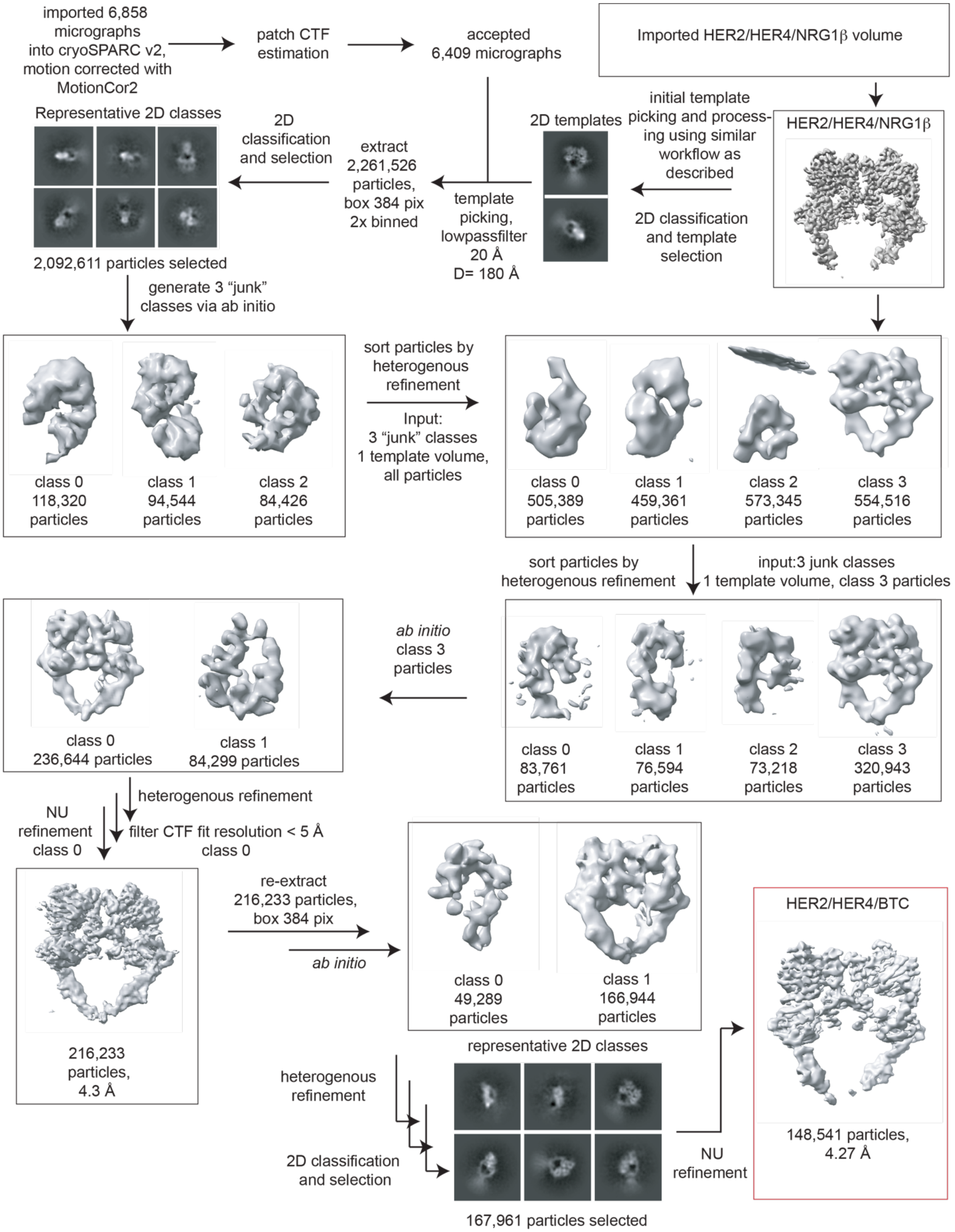

Figure S5. **Processing workflow for the HER2/HER4/BTC structure**. Data were processed in cryoSPARC v2 using a strategy in which particles are picked generously using template picker,

bioRxiv preprint doi: https://doi.org/10.1101/2023.10.06.561161; this version posted January 4, 2024. The copyright holder for this preprint (which was not certified by peer review) is the author/funder, who has granted bioRxiv a license to display the preprint in perpetuity. It is made available under aCC-BY 4.0 International license.

selected by 2D classification to remove bad picks (<10% of particles) and then sorted via 2 rounds of heterogenous refinement into a HER receptor dimer template volume and 3 “junk” classes created from the impure particle stack. Picked particles were subjected to *ab initio* reconstruction to eliminate bias and further processed as shown.

bioRxiv preprint doi: https://doi.org/10.1101/2023.10.06.561161; this version posted January 4, 2024. The copyright holder for this preprint (which was not certified by peer review) is the author/funder, who has granted bioRxiv a license to display the preprint in perpetuity. It is made available under aCC-BY 4.0 International license.

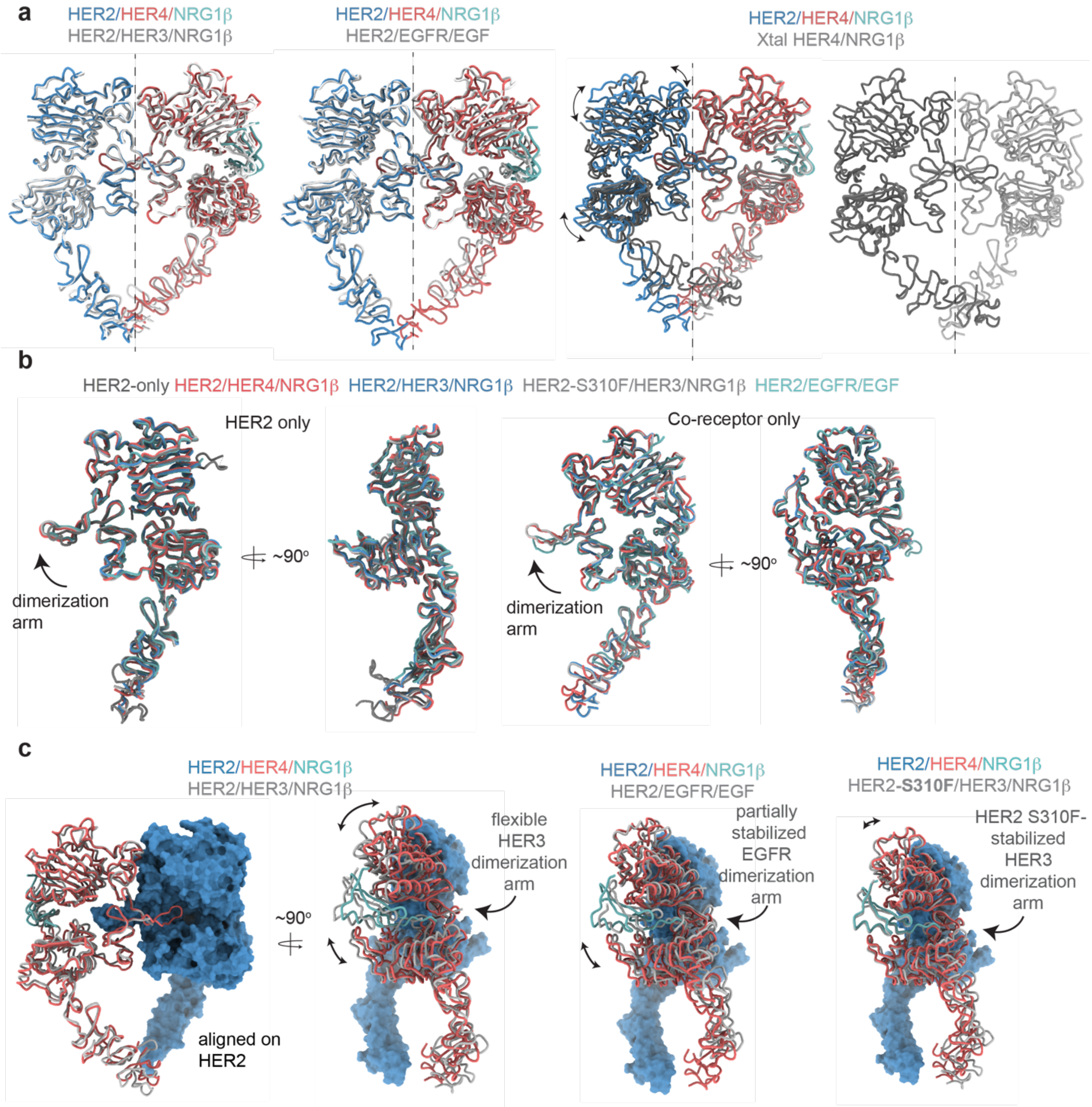

Figure S6. Comparison between the HER2 and HER4 homo- and heterodimeric ectodomain structures. a, Overlays of indicated homo- and heterodimers. Heterodimer alignments were performed using the HER2 chain, alignments with HER4 homodimers were performed using the HER4 chain. The dotted line represents a C2 symmetry axis highlighting the asymmetry of heterodimers compared to near-perfect C2 symmetry observed for HER4/NRG1β homodimers. **b**, Individual receptors from the HER2-containing heterodimers were aligned using the HER2 chain or its co-receptor chain, as indicated. HER2-only is cryo-EM structure of the HER2 ECD with Pertuzumab and Trastuzumab Fab bound (PDB: 6OGE; Fabs not shown),

bioRxiv preprint doi: https://doi.org/10.1101/2023.10.06.561161; this version posted January 4, 2024. The copyright holder for this preprint (which was not certified by peer review) is the author/funder, who has granted bioRxiv a license to display the preprint in perpetuity. It is made available under aCC-BY 4.0 International license.

HER2/HER3/NRG1β (PDB: 7MN5), HER2-S310F/HER3/NRG1β (PDB: 7MN6), HER2/EGFR/EGF (PDB: 8HGO). **c**, Comparison between indicated HER2 heterodimer structures. Structural models are overlayed on HER2 to highlight nuances with which HER2 engages its co-receptors. The same PDB codes were used as in **b**.

bioRxiv preprint doi: https://doi.org/10.1101/2023.10.06.561161; this version posted January 4, 2024. The copyright holder for this preprint (which was not certified by peer review) is the author/funder, who has granted bioRxiv a license to display the preprint in perpetuity. It is made available under aCC-BY 4.0 International license.

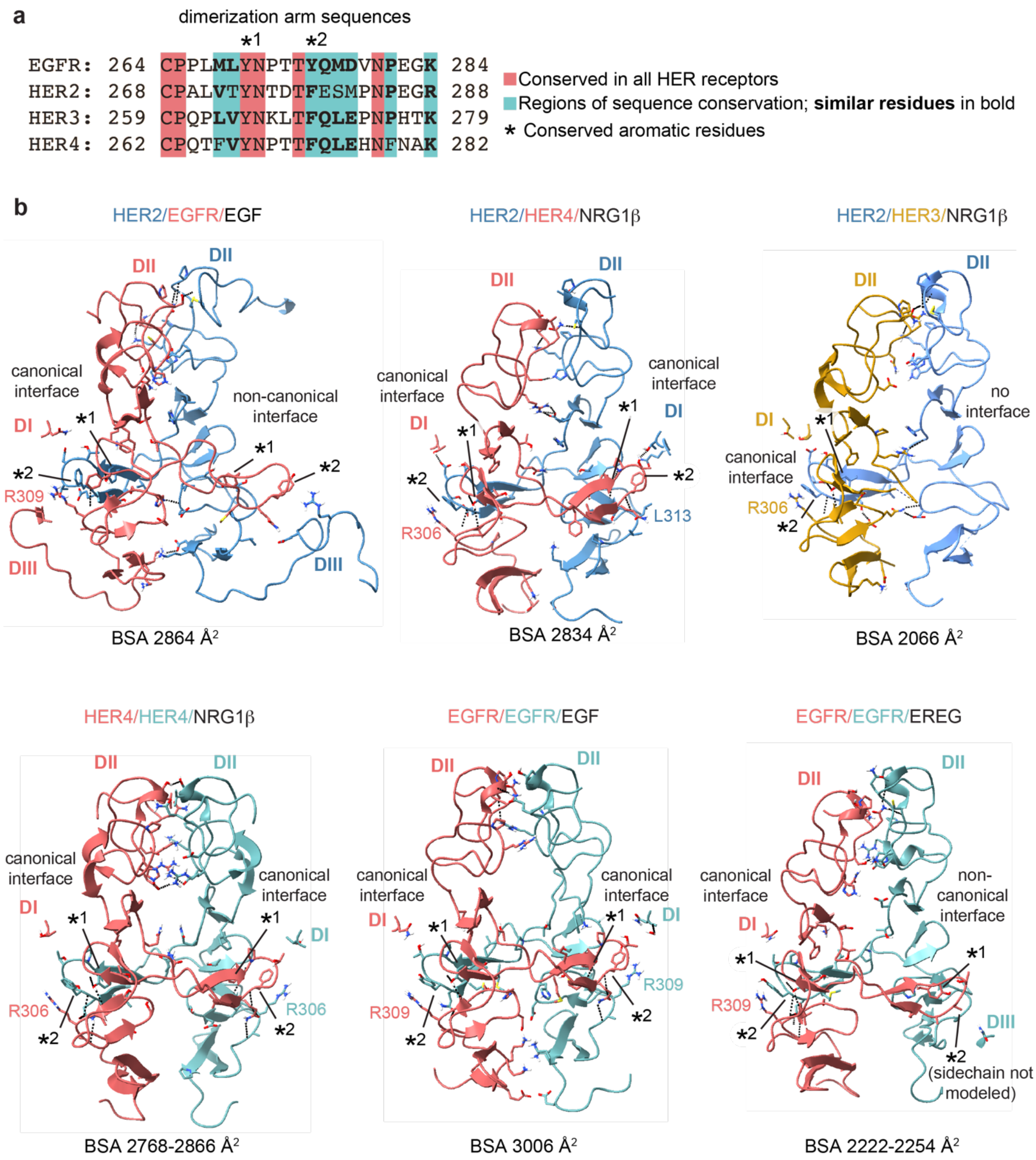

Figure S7. Detailed view of the dimer interfaces of HER receptor homo- and heterodimers. **a**, Sequence alignment of HER receptor dimerization arm regions with conserved residues highlighted in red. Two aromatic residues that are known to engage in hydrogen bonding with the partner receptor are marked with (*). **b**, Full domains II (**DII**) for selected receptor dimers are shown in cartoon and all interface residues between two receptors within domains I and III (**DI-DIII**) are shown as sticks. Hydrogen bonds are indicated with dotted lines. Analysis was performed

bioRxiv preprint doi: https://doi.org/10.1101/2023.10.06.561161; this version posted January 4, 2024. The copyright holder for this preprint (which was not certified by peer review) is the author/funder, who has granted bioRxiv a license to display the preprint in perpetuity. It is made available under aCC-BY 4.0 International license.

using UCSF ChimeraX. Domains IV are not resolved in most structures and are not included in this analysis. Canonical dimerization arm interactions involve domains DI and DII, while non-canonical interfaces, as seen for EGFR in the HER2/EGFR/EGF dimer and one EGFR/EREG monomer in the EGFR/EREG homodimer, engage DIII instead of DI. The following PDB codes were used: HER2/HER3/NRG1 β (PDB: 7MN5), HER2/EGFR/EGF (PDB: 8HGO), EGFR/EGF (PDB: 3NJP), HER4/NRG1β (PDB: 3U7U) and EGFR/EREG (PDB: 5WB7).

bioRxiv preprint doi: https://doi.org/10.1101/2023.10.06.561161; this version posted January 4, 2024. The copyright holder for this preprint (which was not certified by peer review) is the author/funder, who has granted bioRxiv a license to display the preprint in perpetuity. It is made available under aCC-BY 4.0 International license.

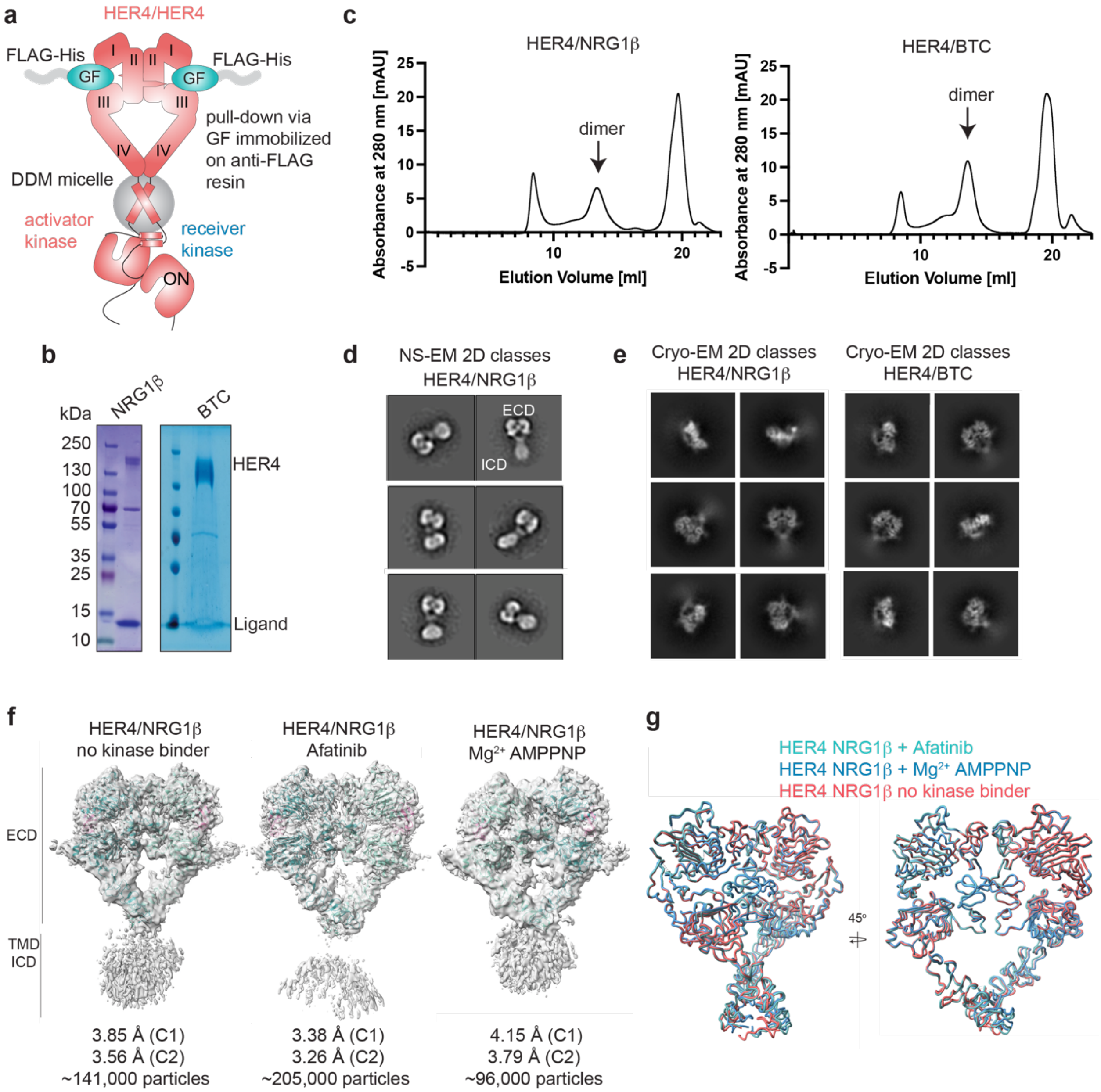

Figure S8. Purification of HER4 homodimers bound to NRG1β or BTC and structural analysis. a, Overview of the HER4 purification strategy. Untagged, full-length HER4 was purified by growth factor (GF)-coated anti-FLAG resin. **b**, Coomassie-stained SDS-PAGE gel showing receptor samples after ligand-mediated receptor pulldown. **c**, SEC profiles of samples after ligand-mediated receptor pulldown using a Superose 6 increase 10/300 GL column. Elution fractions consistent with receptor dimers were used for negative-stain EM (NS-EM) and cryo-EM analyses. **d**, HER4/NRG1β NS-EM 2D class averages show receptor dimers with "heart"-shaped ectodomains and additional density for intracellular kinase domains. **e**, HER4/NRG1β and

bioRxiv preprint doi: https://doi.org/10.1101/2023.10.06.561161; this version posted January 4, 2024. The copyright holder for this preprint (which was not certified by peer review) is the author/funder, who has granted bioRxiv a license to display the preprint in perpetuity. It is made available under aCC-BY 4.0 International license.

HER4/BTC cryo-EM 2D class averages show receptor dimers with "heart"-shaped extracellular domains without density for intracellular kinase domains. **f**, Cryo-EM volumes of HER4/NRG1β obtained from HER4/NRG1β preparations in an apo form, with afatinib, or with Mg^{2+}AMPPNP bound. 10 mM afatinib was added to the culture medium during expression, 1 mM Mg^{2+}AMPPNP was added prior to crosslinking with glutaraldehyde (after FLAG elution). Receptors were subjected to SEC and 1 mM Mg^{2+}AMPPNP was again added prior to cryo-EM grid preparation. **g**, Overlay of models for volumes in **f** show all three volumes are identical.

bioRxiv preprint doi: https://doi.org/10.1101/2023.10.06.561161; this version posted January 4, 2024. The copyright holder for this preprint (which was not certified by peer review) is the author/funder, who has granted bioRxiv a license to display the preprint in perpetuity. It is made available under aCC-BY 4.0 International license.

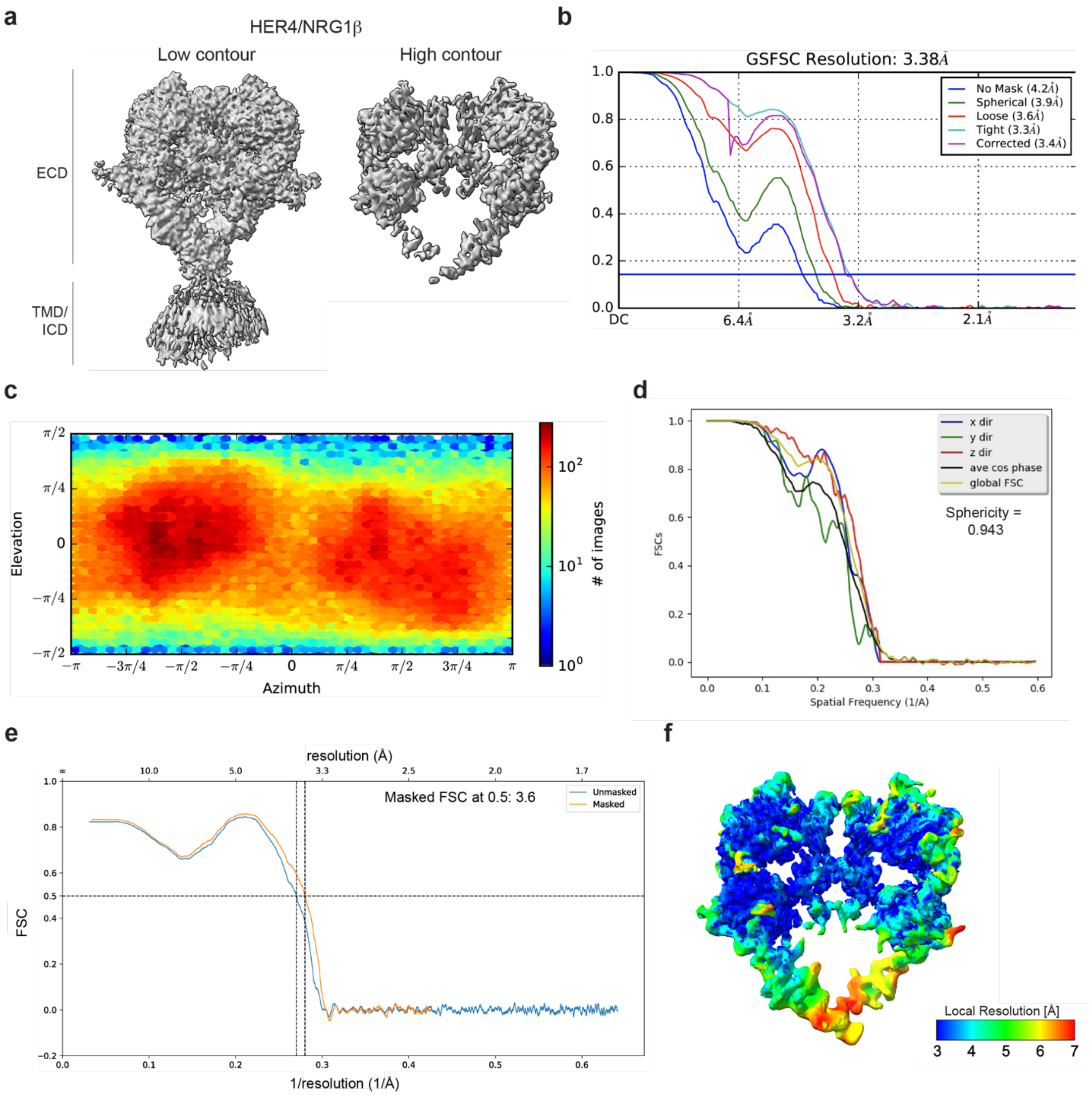

Figure S9. Cryo-EM density maps of HER4 bound to NRG1β processed without symmetry applied. a, Cryo-EM map at different contour levels. **b,** CryoSPARC GSFSC plots. **c**, CryoSPARC Euler angle plots. **d**, 3DFSC plots. **e,** Model-Map-FSC curves from Phenix Validation **f,** Local resolution map of HER4/NRG1β created using cryoSPARC v4

bioRxiv preprint doi: https://doi.org/10.1101/2023.10.06.561161; this version posted January 4, 2024. The copyright holder for this preprint (which was not certified by peer review) is the author/funder, who has granted bioRxiv a license to display the preprint in perpetuity. It is made available under aCC-BY 4.0 International license.

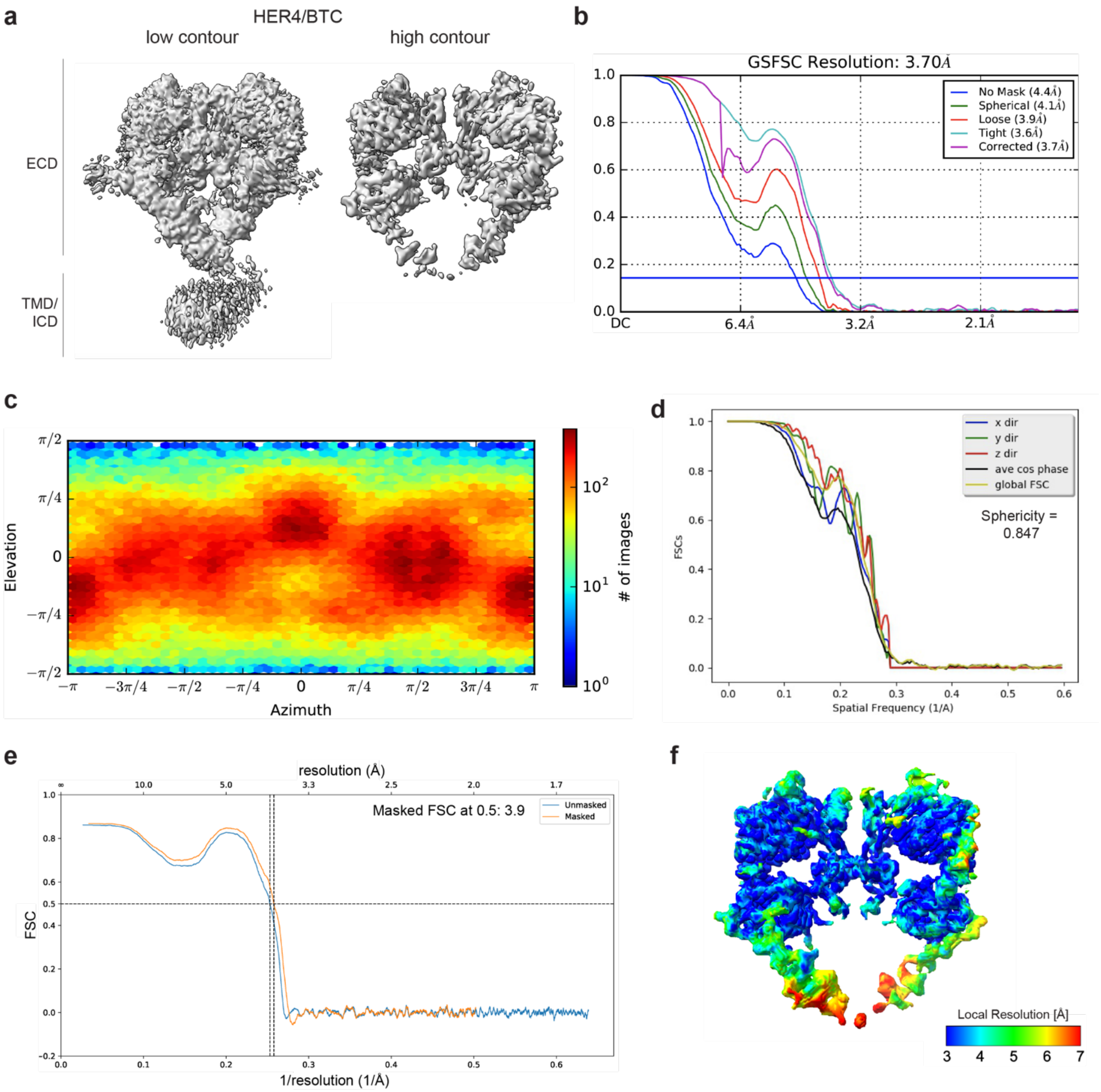

Figure S10. Cryo-EM density maps of HER4 bound to BTC processed without symmetry applied. a, Cryo-EM map at different contour levels. **b,** CryoSPARC GSFSC plots. **c**, CryoSPARC Euler angle plots. **d**, 3DFSC plots. **e,** Model-Map-FSC curves from Phenix Validation **f,** Local resolution map of HER4/BTC created using cryoSPARC v4

bioRxiv preprint doi: https://doi.org/10.1101/2023.10.06.561161; this version posted January 4, 2024. The copyright holder for this preprint (which was not certified by peer review) is the author/funder, who has granted bioRxiv a license to display the preprint in perpetuity. It is made available under aCC-BY 4.0 International license.

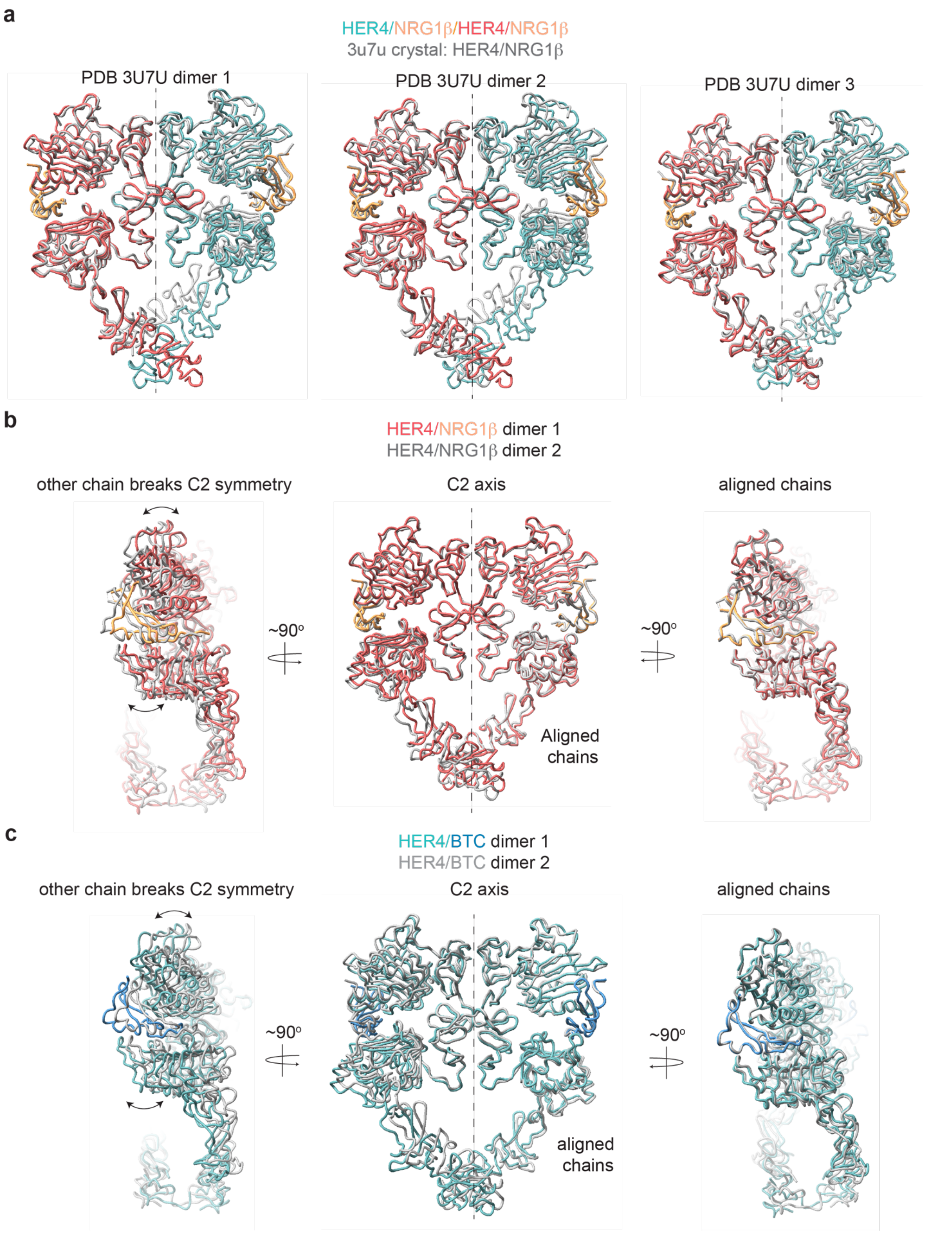

bioRxiv preprint doi: https://doi.org/10.1101/2023.10.06.561161; this version posted January 4, 2024. The copyright holder for this preprint (which was not certified by peer review) is the author/funder, who has granted bioRxiv a license to display the preprint in perpetuity. It is made available under aCC-BY 4.0 International license.

Figure S11. HER4 homodimers do not show ideal C2 symmetry a, Overlay of three HER4/NRG1β ectodomain homodimers found in the asymmetric unit of the crystal structure (PDB: 3U7U) with the cryo-EM structure of full-length HER4/NRG1β. The crystal structure models are shown in grey. RMSDs for overlay of full dimers with the cryo-EM HER4/NRG1β dimer are 5.438 Å, 5.435 Å and 3.662 Å, respectively. **b,** HER4/NRG1β model built into C1 refined cryo-EM map was aligned across chains (chain A in one model aligned to chain B in another model). While the aligned chain showed a near perfect match (RMSD 1.42 Å), the other chain showed breaking of C2 symmetry. **c**, HER4/BTC model built into C1 refined cryo-EM map was aligned across chains (chain A in one model aligned to chain B in another model). While the aligned chain showed a near perfect match (RMSD 1.58 Å), the other chain showed breaking of C2 symmetry.

bioRxiv preprint doi: https://doi.org/10.1101/2023.10.06.561161; this version posted January 4, 2024. The copyright holder for this preprint (which was not certified by peer review) is the author/funder, who has granted bioRxiv a license to display the preprint in perpetuity. It is made available under aCC-BY 4.0 International license.

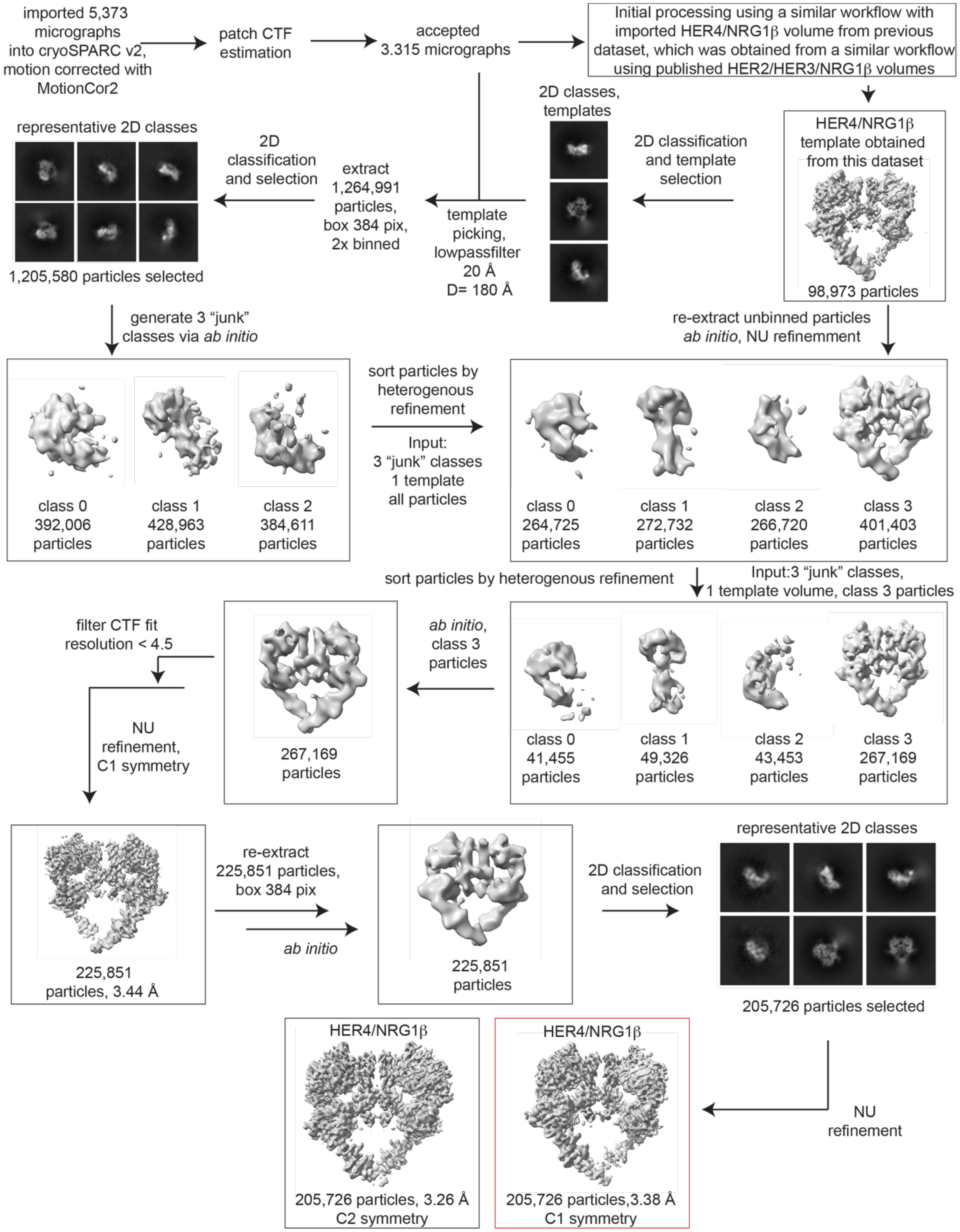

Figure S12. Processing workflow and data statistics for the HER4/NRG1β homodimer. Data were processed in cryoSPARC v2 using a strategy in which particles are picked generously using

bioRxiv preprint doi: https://doi.org/10.1101/2023.10.06.561161; this version posted January 4, 2024. The copyright holder for this preprint (which was not certified by peer review) is the author/funder, who has granted bioRxiv a license to display the preprint in perpetuity. It is made available under aCC-BY 4.0 International license.

template picker, selected by 2D classification to remove bad picks (<10% of particles) and then sorted via 2 rounds of heterogenous refinement into a HER receptor dimer template volume and 3 “junk” classes created from the impure particle stack. Picked particles were subjected to *ab initio* reconstruction to eliminate bias and further processed as shown.

bioRxiv preprint doi: https://doi.org/10.1101/2023.10.06.561161; this version posted January 4, 2024. The copyright holder for this preprint (which was not certified by peer review) is the author/funder, who has granted bioRxiv a license to display the preprint in perpetuity. It is made available under aCC-BY 4.0 International license.

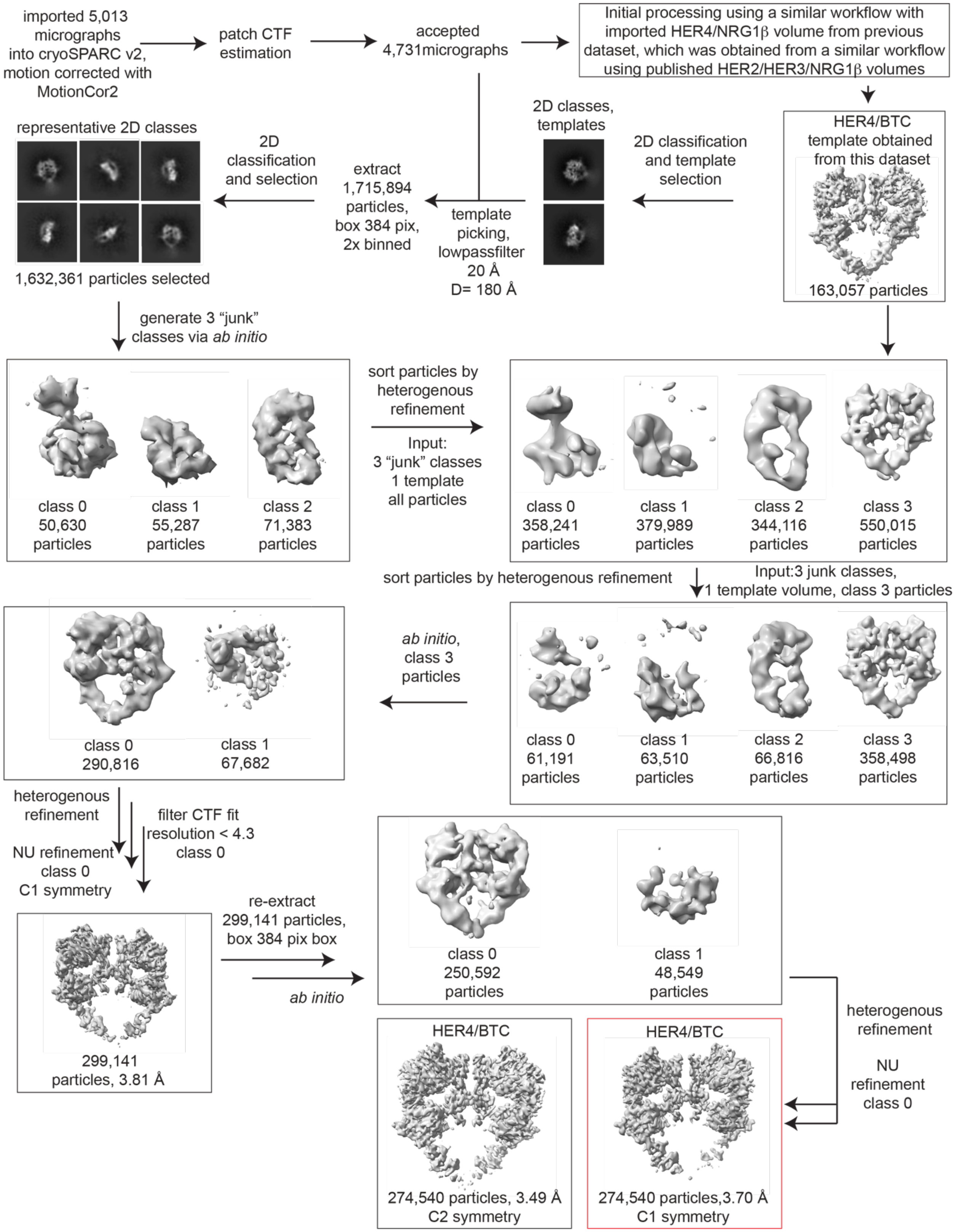

Figure S13. Processing workflow and data statistics for the HER4/BTC homodimer. Data were processed in cryoSPARC v2 using a strategy in which particles are picked generously using

bioRxiv preprint doi: https://doi.org/10.1101/2023.10.06.561161; this version posted January 4, 2024. The copyright holder for this preprint (which was not certified by peer review) is the author/funder, who has granted bioRxiv a license to display the preprint in perpetuity. It is made available under aCC-BY 4.0 International license.

template picker, selected by 2D classification to remove bad picks (<10% of particles) and then sorted via 2 rounds of heterogenous refinement into a HER receptor dimer template volume and 3 “junk” classes created from the impure particle stack. Picked particles were subjected to *ab initio* reconstruction to eliminate bias and further processed as shown.

bioRxiv preprint doi: https://doi.org/10.1101/2023.10.06.561161; this version posted January 4, 2024. The copyright holder for this preprint (which was not certified by peer review) is the author/funder, who has granted bioRxiv a license to display the preprint in perpetuity. It is made available under aCC-BY 4.0 International license.

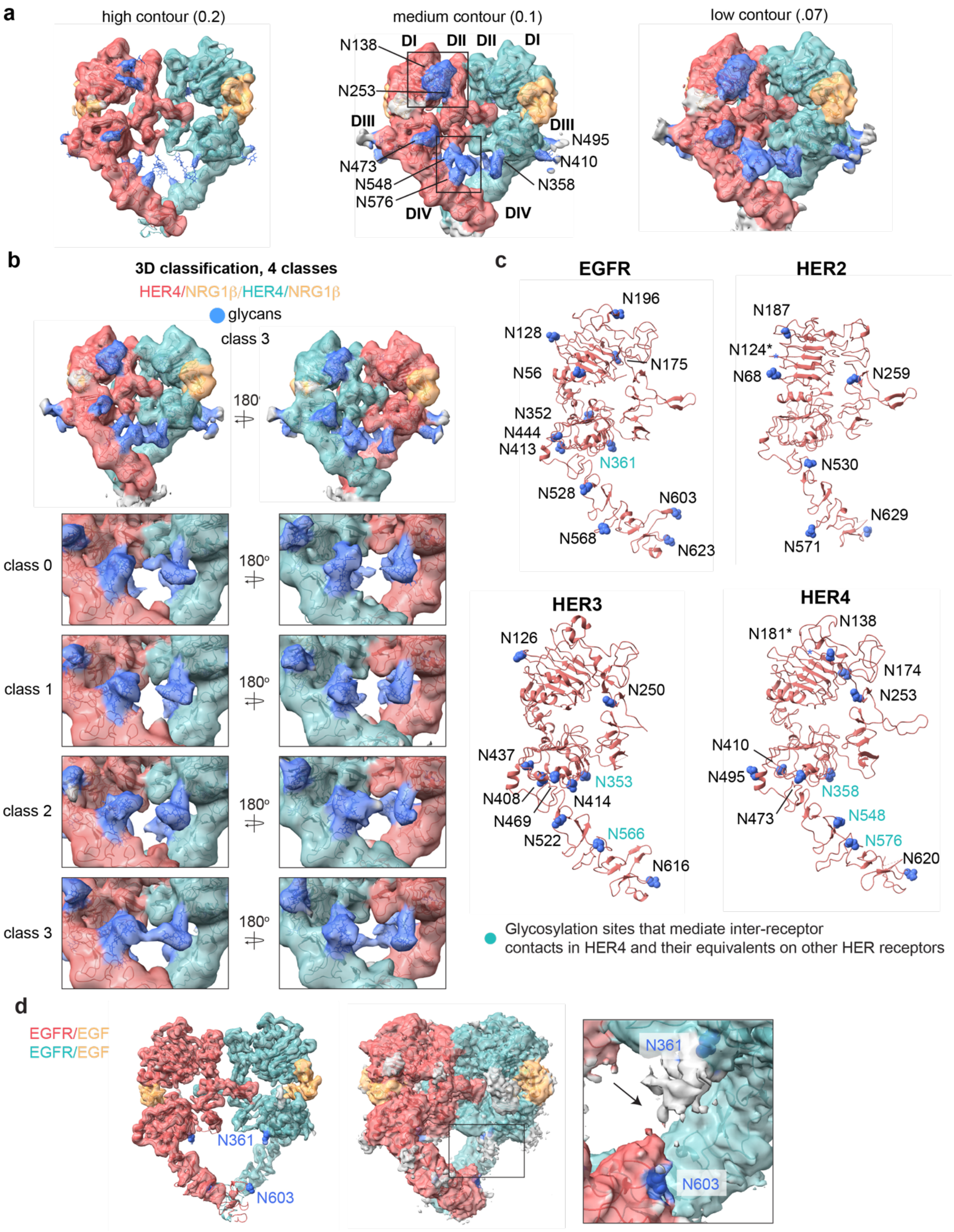

bioRxiv preprint doi: https://doi.org/10.1101/2023.10.06.561161; this version posted January 4, 2024. The copyright holder for this preprint (which was not certified by peer review) is the author/funder, who has granted bioRxiv a license to display the preprint in perpetuity. It is made available under aCC-BY 4.0 International license.

Figure S14. Glycosylation in the cryo-EM structures of HER4/NRG1β homodimer and comparison to other HER receptors. a, Model of the HER4/NRG1β homodimer fitted into the cryo-EM density, lowpass-filtered to 6 Å, at various contour levels. Glycans are shown in blue. **a**, 3D classification of the HER4/NRG1β particles reveals strong continuous glycan density between two receptors within the dimer for class 3. **c**, Glycosylation site asparagines in EGFR, HER2, HER3 and HER4 are marked in blue, and shown in sphere representation. Glycosylation site asparagines involved in inter-receptor contacts in our HER4 structures, and the equivalent residues in other HER receptors are indicated by teal labels. **d**, Analysis of the cryo-EM map of the EGFR/EGF homodimer structure (PDB: 7SYD) at various contour levels suggests the presence of an inter-receptor glycan connection between N353 and N603, shown in blue.
